# Activated Carbon-Decorated Spherical Silicon Nanocrystal Composites Synchronously-Derived from Rice Husks for Anodic Source of Lithium-Ion Battery

**DOI:** 10.3390/nano9071055

**Published:** 2019-07-23

**Authors:** Sankar Sekar, Abu Talha Aqueel Ahmed, Akbar I. Inamdar, Youngmin Lee, Hyunsik Im, Deuk Young Kim, Sejoon Lee

**Affiliations:** 1 Department of Semiconductor Science, Dongguk University-Seoul, Seoul 04620, Korea; 2 Quantum-Functional Semiconductor Research Center, Dongguk University-Seoul, Seoul 04620, Korea

**Keywords:** silicon nanocrystal, activated carbon, nanocomposite, lithium-ion battery

## Abstract

The nanocomposites of activated-carbon-decorated silicon nanocrystals (AC<nc-Si>AC) were synchronously derived in a single step from biomass rice husks, through the simple route of the calcination method together with the magnesiothermic reduction process. The final product, AC<nc-Si>AC, exhibited an aggregated structure of activated-carbon-encapsulated nanocrystalline silicon spheres, and reveals a high specific surface area (498.5 m^2^/g). Owing to the mutualization of advantages from both silicon nanocrystals (i.e., low discharge potential and high specific capacity) and activated carbon (i.e., high porosity and good electrical conductivity), the AC<nc-Si>AC nanocomposites are able to play a substantial role as an anodic source material for the lithium-ion battery (LIB). Namely, a high coulombic efficiency (97.5%), a high discharge capacity (716 mAh/g), and a high reversible specific capacity (429 mAh/g after 100 cycles) were accomplished when using AC<nc-Si>AC as an LIB anode. The results advocate that the simultaneous synthesis of biomass-derived AC<nc-Si>AC is beneficial for green energy-storage device applications.

## 1. Introduction

The lithium-ion batteries (LIBs) have garnered global interest because of its high energy capacity, low toxicity, long lifetime, high rate capability, and no memory effect. Due to their ample popularity in various applications (e.g., electric vehicles and portable electronic systems) [1,2,3,4], high-power LIBs have been of great interest as next-generation energy-storage devices. The electrochemical performance of LIBs is closely related to the choice of anode material; thus, various anode materials (e.g., Si, C, Sn, NiO, VO_x_ etc.) have been studied so as to improve the LIB characteristics. Among them, Si is one of the prominent candidates as a LIB anodic source, because Si has extraordinary properties of a high specific capacity (~4200 mAh/g for Li_22_Si_5_) and a low discharge potential (~0.1 V per Li/Li^+^) [5,6,7,8,9,10,11]. The Si-based LIB anode, however, still suffers from a critical issue of tremendous volume change (≥300%) after multiple processes of Li alloying and de-alloying. This causes both the pulverization and the fracture of the Si lattices, and creates a metastable solid–electrolyte interphase (SEI) layer. The continual formation of such an SEI layer tends to degrade the energy capacity and the cyclic stability of the LIBs [12,13,14,15,16,17,18]. To reduce this drawback, it has been proposed that the structure of Si be modified into the nanoscale and used to form several types of composite material mediums [15,16,17,18,19,20,21,22,23]. For instance, Si nanoarchitectures (e.g., nanoparticles, nanopores, hollow nanospheres, nanotubes, and nanowires) have been known to significantly reduce physical strain, volume change, and mechanical fracture loss [24,25,26,27,28,29]. Although there are several methods to prepare Si nanostructures (e.g., chemical vapor deposition [30], chemical doping [31], wet etching [32], and laser ablation [33,34]), a simple and cheap approach is still fascinating.

Meanwhile, the derivation of electrode materials from recyclable, earth-abundant, eco-friendly substances is one of the pivotal steps required for the design of green technology. Since agricultural biomass wastes contain a large percentage of inorganic constituents, several kinds of electrode materials have been derived from various biomass resources. For example, rice husks are one of the most impressive siliceous and carbonaceous precursors for producing silica [35,36,37], silicon [38,39,40], silicon tetrachloride [41], silicon nitride [42], silicon carbide [43], graphene [44,45,46], and activated carbon (AC) [47]. Due to the advantages of both silicon nanoarchitectures (i.e., low discharge potential and high specific capacity) and carbon nanostructures (i.e., high porosity and good electrical conductivity), there have been several attempts to derive both silicon- and carbon-based materials from a single biomass resource of rice husks [20,48,49]. However, the simultaneous derivation of Si-AC nanocomposites from rice husks through a single synthesis process has rarely been reported so far, despite the synergetic effects of both Si and AC on the electrochemical performances of the LIBs.

Herein, we report the synchronous derivation of Si-AC nanocomposites from a single biomass resource via a single-step simultaneous synthesis method. High quality, AC-decorated Si nanocrystals (AC<nc-Si>AC) are derived from brown rice husks (BRHs) through a simple process of magnesiothermic reduction. In comparison with other techniques, our method is very beneficial for the following reasons: (i) the whole process is time- and energy-efficient, (ii) the entire process does not require commercial sources of carbonaceous and siliceous precursors, and (iii) the final products of AC<nc-Si>AC nanocomposites have a unique structure that can enhance LIB performance. To identify the physical and electrochemical properties of the BRH-derived AC<nc-Si>AC nanocomposites, we also show and compare the material characteristics of nc-Si and AC that were synthesized from same BRHs.

## 2. Experimental

### 2.1. Synthesis of AC-Decorated nc-Si

**Figure 1:**
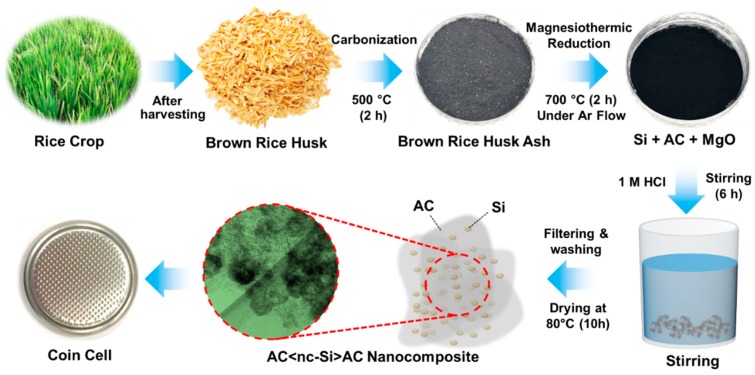
Experimental procedures for the synchronous derivation of activated-carbon-decorated silicon nanocrystal (AC<nc-Si>AC) nanocomposites from the biomass resource of brown rice husks (BRHs).

The AC<nc-Si>AC nanocomposites were synthesized using BRHs (produced in Tamil Nadu, India) through the magnesiothermic reduction process (Figure 1). To collect the ashes of BRHs, BRHs were first annealed in air at 500 °C for 120 min. We then mixed 1.0 g of BRH ashes with 0.2 g of Mg powder. The BRH-Mg mixtures were transferred into an alumina crucible, and were subsequently annealed in Ar at 700 °C for 120 min. During this step, the constituents of AC<nc-Si>AC, Mg_2_Si, and MgO could be produced through magnesiothermic reduction via the following reaction:

$$2\mathrm{Mg}+{\mathrm{SiO}}_{2}/C\;\left(\mathrm{BRH}\;\mathrm{ashes}\right)\underset{700℃}{\to }2\mathrm{MgO}+\mathrm{AC}\langle \mathrm{nc}-\mathrm{Si}\rangle \mathrm{AC}.$$

To extract AC<nc-Si>AC nanocomposites and to remove Mg_2_Si precipitates, MgO residues, and naive SiO_2_ impurities, the annealed mixtures were stirred for 6 h in 1 M HCl, and were reacted with HF (5%) for 1 h. Thereafter, the reacted solution was filtered and rinsed in deionized water to collect the wet products of AC<nc-Si>AC. Finally, the resultant products were dried in vacuum for 10 h at 80 °C. To identify the material characteristics of the BRH-derived AC<nc-Si>AC nanocomposites, we also prepared and characterized nc-Si and AC from the same biomass resource of BRHs (See Supplementary Materials for the experimental procedures used for nc-Si and AC.).

### 2.2. Characterization of Material Properties

The morphological properties of nc-Si, AC, and AC<nc-Si>AC were examined by scanning electron microscopy (SEM, Phillips, Eindhoven, The Netherlands). In addition, the microstructures were also monitored by transmission electron microscopy (TEM, JEOL USA Inc. Peabody, MA, USA) and in situ selective-area electron diffractometry (SAED). The structural and vibrational properties were examined by x-ray diffractometry (XRD, Bruker, Madison, WI, USA) and Raman scattering spectroscopy (HORIBA Jobin Yvon Inc. Edison, NJ, USA), respectively. The pore characteristics were assessed using nitrogen adsorption–desorption isotherm (N_2_ ADI, MicrotracBEL, Osaka, Japan) measurements.

### 2.3. Analyses of Electrochemical Performances

The electrochemical performances of nc-Si, AC, and AC<nc-Si>AC as the LIB anodes were measured utilizing a 2032 type coin cell with a separator of Celgard PP/PE/PP and a counter electrode of Li foil. To fabricate the LIB anodes, firstly, we first prepared three different types of slurries by mixing a carbon black (10 wt.%) + polyvinylidene difluoride (10 wt.%) mixture with three different active materials, namely nc-Si (80 wt.%), AC (80 wt.%), or AC<nc-Si>AC (80 wt.%), in N-methyl-2-pyrrolidinone solution. Each slurry product was casted on the Cu foil, and each sample was subsequently cured in a vacuum at 110 °C for 5 h. The 2032 type coin cells with three different active materials were then fabricated under Ar (99.999%) ambience in a glove box. Here, we used an electrolyte solution mixed with 1 M LiPF_6_ solvent with an 1: 1 mixture of OC(OCH_3_)_2_ and (CH_2_O)_2_CO. The cyclic voltammetry (CV) and the galvanostatic charge–discharge (GCD) characteristics were evaluated at a potential widow of 0.01–3.0 V (vs. Li/Li^+^). Finally, the charge-transfer impedance characteristics were assessed by electrochemical impedance spectrometry (EIS) measurements at frequencies ranging from 0.01 Hz to 100 kHz.

## 3. Results and Discussion

### 3.1. Morphological Properties

**Figure 2:**
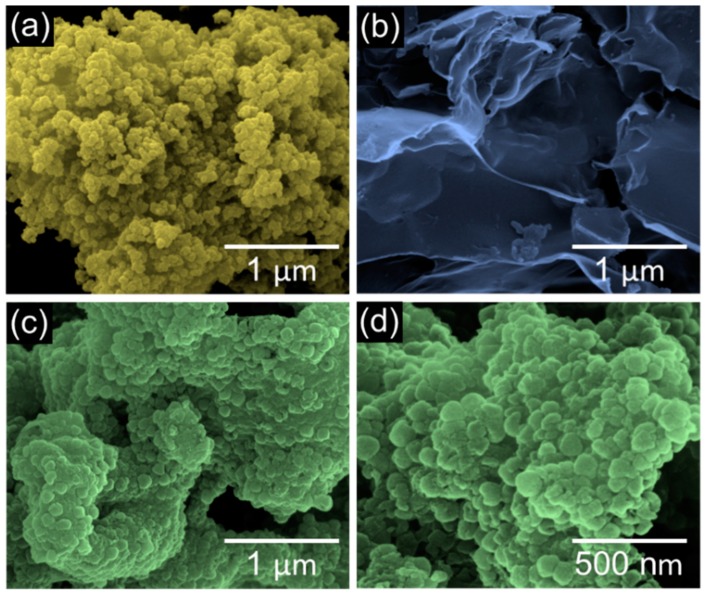
Scanning electron microscope (SEM) images of (**a**) silicon nanocrystals (nc-Si), (**b**) activated carbon (AC), (**c**) AC<nc-Si>AC nanocomposites, and (**d**) magnified SEM image of the AC<nc-Si>AC nanocomposites.

**Figure 3:**
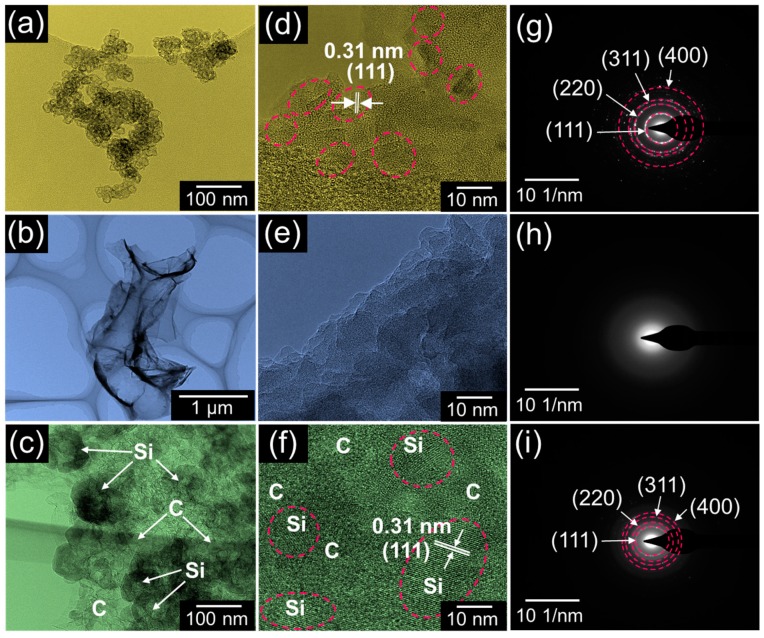
Bright-field transmission electron microscope (TEM) images of (**a**) nc-Si, (**b**) AC, and (**c**) AC<nc-Si>AC nanocomposites. High-resolution TEM images of (**d**) nc-Si, (**e**) AC, and (**f**) AC<nc-Si>AC nanocomposites. Selective-area electron diffractometry (SAED) patterns (**g**) nc-Si, (**h**) AC, (**i**) AC<nc-Si>AC nanocomposites.

The morphological properties of the BRH-derived nc-Si, AC, and AC<nc-Si>AC were investigated by SEM. In the case of nc-Si (Figure 2a), a lot of spherical Si nanocrystals (15–20 nm) were interconnected in the form of densely-aggregated nc-Si bundles. The interconnected morphology of spherical nc-Si is of benefit for promoting electrochemical action in LIB devices because of both the high specific capacity [5,6,7,8,9] and the minimal change in surface-to-volume ratio during the ion storage process [50]. On the other hand, AC displayed a nanosheet-like morphology (Figure 2b), and each AC nanosheet was spread along the surface direction. This two-dimensional nature of AC is favorable for enhancing both ion diffusion and ion storage in LIBs [51,52]. Similarly to nc-Si, AC<nc-Si>AC showed an aggregated structure of spherical nanoparticles with the increased diameter of 40–60 nm (Figure 2c,d). The increase in the particle size is relevant to wrapping of Si nanocrystals with AC nanosheets (see also Figure 3c). From the energy dispersive x-ray analysis, nc-Si, AC, and AC<nc-Si>AC were confirmed to possess their main species of Si, C, and Si-C, respectively (Supplementary Materials), which means that the samples were effectively synthesized from the biomass siliceous and carbonaceous resource of BRHs.

### 3.2. Microstructural Properties

To glean further insights into the microstructures of the samples, we performed TEM and SAED measurements. For the BRH-derived nc-Si (Figure 3a), it was confirmed that the Si nanoparticles had a regular shape of nanospheres with an average diameter of ~18 nm. As indicated in Figure 3d, the spacing between the interlayer fringes was ~0.31 nm, and this value was consistent with that of the Si (111) plane. Additionally, the SAED pattern of nc-Si revealed a regular array of diffraction patterns, spotted along with four well-placed diffraction rings (Figure 3g). These verified that the BRH-derived nc-Si had a crystalline nature. In the case of AC (Figure 3b), the sheet-like two-dimensional morphology was clearly observable. As can be seen from Figure 3e,h, the AC nanosheets were amorphous. In Figure 3c, the microstructure of AC<nc-Si>AC is shown. The sample clearly exhibited a nanocomposite morphology that comprised spherical Si nanocrystals embedded into the AC nanosheets. Furthermore, the high-resolution TEM image also shows the Si nanocrystals to be wrapped with amorphous AC carbon nanosheets (Figure 3f). As shown in the SAED pattern (Figure 3i), both the amorphous phase of AC and the crystalline phase of nc-Si simultaneously appeared. This implies that the magnesiothermic reduction method is useful for the synchronous derivation of the AC<nc-Si>AC nanocomposites from biomass resource of BRHs.

### 3.3. Crystallographic Characteristics

**Figure 4:**
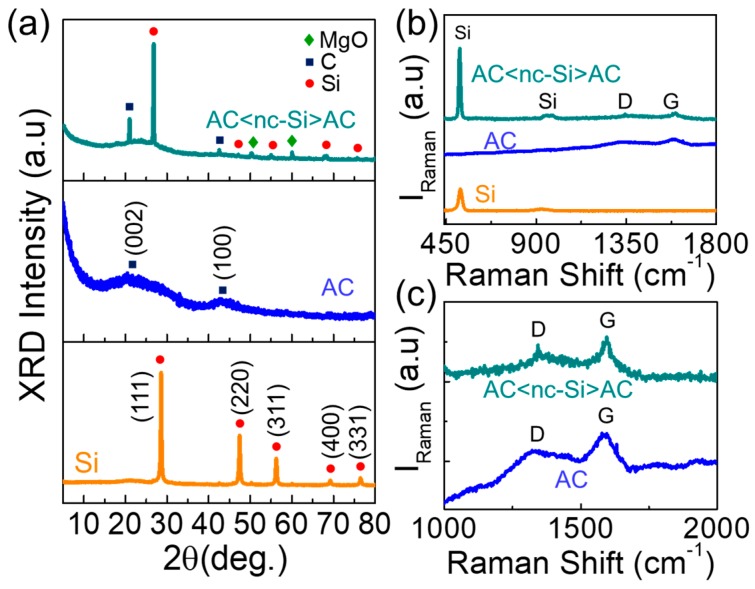
(**a**) X-ray diffractometry (XRD) patterns and (**b**) Raman spectra of nc-Si, AC, and AC<nc-Si>AC nanocomposites. (**c**) Highlighted view of the Raman spectra for AC and AC<nc-Si>AC nanocomposites.

The crystallographic characteristics of nc-Si, AC, and AC<nc-Si>AC were investigated through XRD measurements (Figure 4a). From nc-Si, five diffraction patterns were observed at 26.7°, 47.1°, 55.1°, 68.1°, and 75.8°, corresponding to (111), (220), (311), (400), and (331) lattice planes of crystalline Si (JCPDS no. 27-1402) [18,53], respectively. In addition, AC displayed the typical activated-carbon phases of (002) and (100) at 21.2° and 42.6°, respectively [54,55,56]. As can be confirmed from Figure 4a, the diffraction patterns of AC<nc-Si>AC belonged to those of nc-Si and AC, representing an effective formation of a nanocomposite structure consisting of both nc-Si and AC. Two small MgO peaks were thought to come from MgO residues. The formation of the nanocomposite structure can also be confirmed from its Raman scattering characteristics. As displayed in Figure 4b, the AC<nc-Si>AC nanocomposites clearly showed Raman scattering features from both Si and AC, but revealed no MgO peaks. The absence of MgO-related peaks indicates that a small amount of MgO residue might be covered by some parts of nc-Si and AC during the HCl treatment. Namely, the Raman bands at ~519 cm^−1^ and ~966 cm^−1^ arose from crystalline Si [18,19], and the D (~1343 cm^−1^) and G (~1596 cm^−1^) bands originated from graphitized carbon [57,58] (see also Figure 4c).

### 3.4. Textural Characteristics

**Figure 5:**
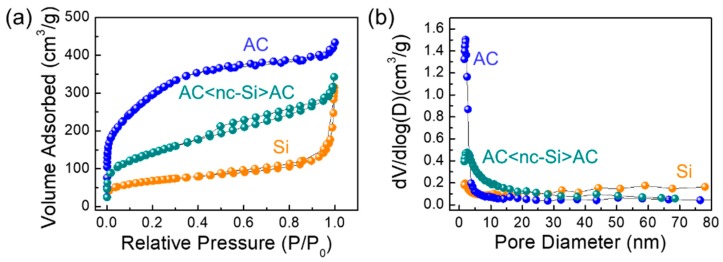
(**a**) Nitrogen adsorption–desorption isotherm (N_2_ ADI) characteristics and (**b**) pore characteristics of nc-Si, AC, and AC<nc-Si>AC nanocomposites.

The textural characteristics of the samples were evaluated through N_2_ ADI measurements. As shown in Figure 5a, nc-Si and AC<nc-Si>AC exhibited a typical mesoporous characteristic with the type-IV isotherm [59]. However, AC showed a type-H4 hysteresis loop with the type-II/IV bimodal sorption isotherm characteristic, representing a coexistence of micro- (≤2 nm), meso- (2–50 nm), and macro-pores (≥50 nm) in the material [60,61]. Through the Brunauer–Emmett–Teller (BET) analysis, the specific surface areas of nc-Si, AC, and AC<nc-Si>AC were determined to be 232.8, 1068.5 and 498.5 m^2^/g, respectively. From the pore diameter vs. dV/dlog (D) curves (Figure 5b), the pore surface area of nc-Si, AC, and AC<nc-Si>AC were additionally estimated to be 153.5, 755.8, and 394.9 m^2^/g, respectively. Through the Barrett–Joyner–Halenda (BJH) analysis, the average pore diameter and the total pore volume of the Si nanocrystals were calculated to be 5.83 nm and 0.294 cm^3^/g, and those of the AC nanosheets were 2.41 nm and 0.493 cm^3^/g, respectively. Compared to nc-Si, AC<nc-Si>AC had a smaller average pore diameter (~2.45 nm) and a larger total pore volume (~0.386 cm^3^/g) because of the morphological structure of AC-decorated n-Si in AC<nc-Si>AC (see also Table 1).

### 3.5. Electrochemical Performances

**Figure 6:**
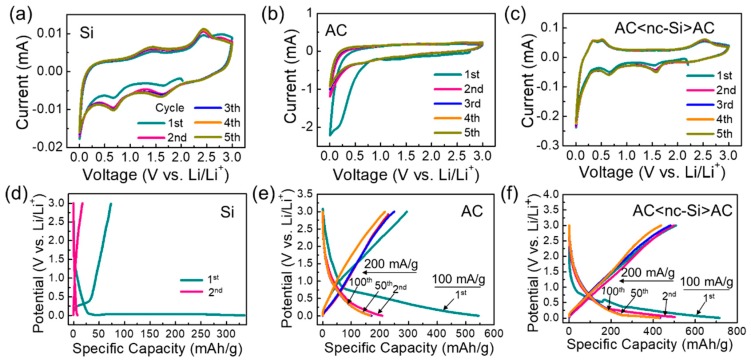
Cyclic voltammetry (CV) curves of the lithium-ion battery (LIB) devices with the (**a**) nc-Si, (**b**) AC, and (**c**) AC<nc-Si>AC nanocomposites as anode materials. All of the CV measurements were conducted under the scanning rate of 0.1 mV/s. Galvanostatic charge–discharge (GCD) curves of the LIB devices with (**d**) nc-Si, (**e**) AC, and (**f**) AC<nc-Si>AC nanocomposites as anode materials. For the first GCD cycle, a current density of 100 mA/g was applied, and the latter cycles were performed at an applied current density of 200 mA/g.

Such a nanocomposite morphology of AC-decorated n-Si (i.e., AC<nc-Si>AC) may improve the electrochemical performance of the LIB device because of the mutualization of advantages from both nc-Si and AC. Therefore, the electrochemical properties of the samples were assessed after fabricating LIB anode structures using nc-Si, AC, and AC<nc-Si>AC. First, the CV characteristics of the fabricated anodes were measured at 0–3.0 V (vs. Li/Li^+^) with a scan rate of 0.1 mV/s. In the LIB sample with the nc-Si anode (Figure 6a), the anodic peaks clearly appeared at ~1.4 and ~2.5 V, attributed to the intercalation of lithium ions into nc-Si [62]. Additionally, a cathodic peak appeared at ~0.66 V during the first CV cycle, which might be due to both the electrolyte decomposition and the SEI layer formation on the electrode surface [63]. Since the SEI film restricts further dissolution of the electrolyte and improves the LIB stability [39], the subsequent CV curves were stabilized during the second–fifth cycles. A broad cathodic peak at ~1.65 V was thought to result from the reaction of Li ions with native SiO_2_ residing at the nc-Si surface [64,65]. In the case of the AC anode (Figure 6b), a typical cathodic peak at ~0.2 V was observable for the first CV cycle. The subsequent CV curves became stable, presumably due to the formation of the SEI layer onto the AC surface [55,66,67,68]. When using AC<nc-Si>AC as an anodic source for the LIB device (Figure 6c), similarly to the case of nc-Si, the cathodic peaks were visible at ~0.66 and ~1.65 V. Furthermore, the device revealed two additional peaks at 0.33 and 0.52 V, attributed to the formation of Li_x_Si and Li_x_C during the lithiation of Si and C with Li ions [14,69,70]. However, no MgO-related peaks were observed from the device, although the nanocomposites involved a small amount of MgO residue. Compared to the nc-Si case, the anodic reactions of the AC<nc-Si>AC nanocomposites were very strong. Hence, one can conjecture that a faster electrochemical reaction occurred in AC<nc-Si>AC, presumably because of the existence of the highly-conductive AC nanosheets in the AC–Si nanocomposite system [71].

The electrochemical performances of the samples were further assessed though GCD measurements. In the case of nc-Si (Figure 6d), the charge (i.e., delithiation) and the discharge (i.e., lithiation) capacities were 336 and 75 mAh/g at the first charge–discharge cycle, respectively. Accordingly, the initial coulombic efficiency reached a low value of ~22%, and this poor efficiency was considered to result from the lithiation of native oxides [17]. The first discharge curve displays a wide plateau at ~0.09 V, indicating the formation of Li–Si alloys and SEI layers on the surface of the anode material [18,19]. In the second cycle, the GCD properties were drastically degraded because of the poor electronic conductivity, slower chemical reaction, and lower surface area [71]. For the case of AC (Figure 6e), the charge and the discharge capacity values were 295 and 545 mAh/g, respectively, yielding a coulombic efficiency of 54%. The initial discharge curve showed a potential plateau at 0.8 V because of the SEI layer formation. However, the plateau promptly disappeared after the subsequent charge–discharge cycle, due to the good stability of the carbonaceous LIB anode [51,72]. When using the AC<nc-Si>AC anode (Figure 6f), the LIB device exhibited a greater charge capacity (510 mAh/g) and a larger discharge capacity (716 mAh/g) than other samples. Accordingly, the AC<nc-Si>AC anode also had a higher coulombic efficiency of 71%. The enhanced efficiency can be interpreted as being due to the configuration of the AC-wrapped nc-Si nanocomposite structure. Namely, such an encapsulation of nc-Si by AC could effectively suppress the undesired reaction (i.e., Li–Si alloying behavior) at the nc-Si surface, while the electron diffusion could be enhanced due to the existence of mesoporous nc-Si [17,73,74]. Three potential plateaus (0.3, 0.5, and 0.8 V) in the first discharge cycle were believed to result from the SEI layer formation and the lithiation of amorphous Li_x_Si and Si [15,16,59].

**Figure 7:**
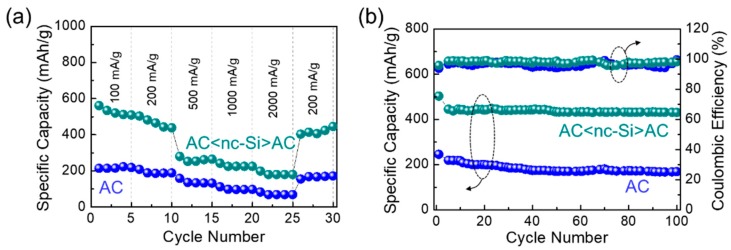
(**a**) Rate performance at various current densities for the LIB devices with the anode materials of AC nanosheets and AC<nc-Si>AC nanocomposites. (**b**) Cycling performance and coulombic efficiency measured under the applied current density of 200 mA/g for the LIB devices with the anode materials of AC and AC<nc-Si>AC nanocomposites.

Next, we examined the rate performance and the cyclic stability of LIBs with the AC and the AC<nc-Si>AC anodes. For this analysis, the LIB device with the nc-Si anode was excluded because of its poor electrical conductivity, as discussed later in detail. Figure 7a shows the dependence of the rate performance on the applied current density. The LIB with the AC anode revealed a reversible discharge capacity from 214 to 83 mAh/g when varying the applied current density from 100 to 2000 mA/g, respectively. Similarly to the above, the LIB with the AC<nc-Si>AC anode delivered a reversible discharge capacity from 561 to 199 mAh/g upon varying the applied current density from 100 to 2000 mA/g, respectively. In both cases, the discharge capacity was effectively recovered when switching the applied current density back to 200 mA/g after 25 cycles. This indicates that both devices had a good reversibility. However, the initial specific capacity (i.e., under the first cycle with 100 mA/g) and the specific capacity window (i.e., deviation of the specific capacity upon varying the applied current density) were approximately 2.6 times and 1.5 times greater for the case of AC<nc-Si>AC, respectively, compared to the case of AC.

Furthermore, the AC<nc-Si>AC anode showed not only an excellent cyclic performance, but also a good coulombic efficiency. As represented in Figure 7b, the AC<nc-Si>AC anode maintained its high discharge capacity (429 mAh/g) over 100 cycles, whereas the AC anode revealed a 0.38 times lower discharge capacity (166 mAh/g) than AC<nc-Si>AC. In addition, the LIB with the AC<nc-Si>AC anode sustained a relatively high coulombic efficiency (~97.5%) during 100 charge–discharge cycles. Compared to commercial graphite (372 mAh/g) [17], the observed discharge capacity was increased by a factor of ~1.2 because of the high specific capacity from Si in AC<nc-Si>AC nanocomposites [5,6,7,8,9]. Both the two-dimensional nature and the high electronic conductivity of the AC nanosheets [16,18] in AC<nc-Si>AC may also enhance ion diffusion and ion storage in the LIBs [18]. Such excellent rate and cyclic performances can be attributed to the good structural stability of the AC<nc-Si>AC nanocomposites [63]. In other words, the AC nanosheets helped to minimize the volumetric change of the Si nanocrystals because the AC nanosheets played a role as an encapsulation layer and/or a shell of the Si nanocrystals. Since the AC shell accommodated a sufficient area for Li ion diffusion for the charge–discharge action, the reaction of Li with nc-Si could be considerably inhibited in the entire solid-state system of the AC<nc-Si>AC nanocomposite. Compared to other state-of-the art Si-based nanocomposites [15,16,17,18,19,20,21,22,23], the capacity retention of AC<nc-Si>AC is comparable or even higher than that of the composites produced by all commercial resources (see Table S1). Even though the initial capacity should be further improved for our fully biomass-derived AC<nc-Si>AC, doping of nc-Si and incorporating highly-conductive nanomaterials (e.g., graphene, carbon nanotube, conducting polymer etc.) can be the next step towards increasing the electrical conductivity and the porosity of the entire composite material system for further improvement of its electrochemical performance.

**Figure 8:**
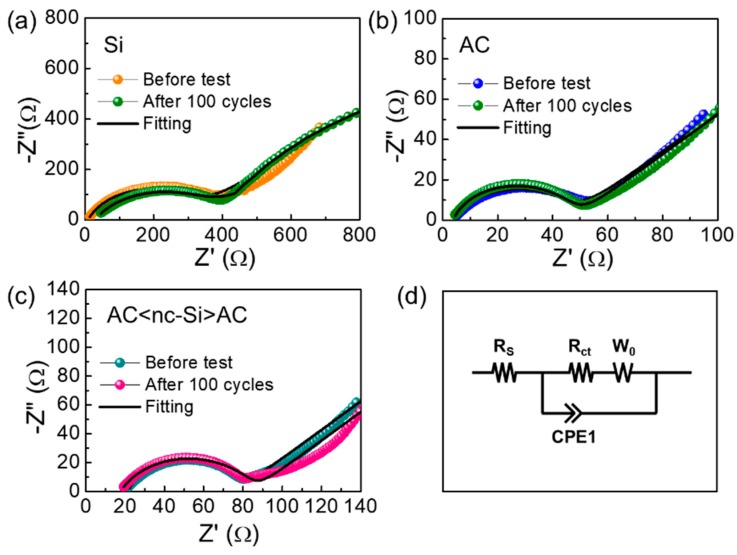
Nyquist plots measured at the discharged state before and after the stability test for the LIB devices with the (**a**) nc-Si, (**b**) AC, and (**c**) AC<nc-Si>AC nanocomposites as anode materials. (**d**) Equivalent circuit used for the fitting analysis of the LIB device.

Finally, we assessed the electrochemical transport properties of the LIBs through the EIS measurements at the discharged state before and after the stability test (i.e., 100 charge–discharge cycles). Figure 8a–c shows the Nyquist plots of the LIB samples with the nc-Si, AC, and AC<nc-Si>AC anodes, respectively. All of the devices exhibited two distinctive features; i.e., one was a semicircle at the high frequency, and the other was a line at the low frequency. The former was attributed to the charge transfer resistance (R_ct_) at the electrolyte–electrode interface [17,75], and the latter was associated with the Warburg impedance (W_Z_), representing Li ion diffusion into the anodic material [18,76]. Through best fitting on the basis of the equivalent LIB circuit (Figure 8d), the R_ct_ values of the nc-Si, AC, and AC<nc-Si>AC anodes were calculated to be 329.8, 42.8, and 56.4 Ω, respectively. The nc-Si sample exhibited a relatively high resistance, leading to a poor electrochemical performance. For this reason, the measurements of rate performance and cyclic stability excluded the nc-Si. In comparison with the nc-Si anode, the AC<nc-Si>AC anode revealed a much lower value of R_ct_ because of the incorporation of highly-conductive AC in the AC<nc-Si>AC nanocomposites. This allowed a faster electron transfer to the electrode–electrolyte interface [77], and it led to a remarkable improvement in anodic performance of AC<nc-Si>AC (i.e., enhanced discharge capacity, increased coulombic efficiency, high rate capability, excellent cyclic stability, etc.).

## 4. Conclusions

Nanocomposites of AC<nc-Si>AC were synchronously derived in a single step from BRHs via the magnesiothermic reduction process. The AC<nc-Si>AC nanocomposites exhibited an interconnected structure of spherical Si nanocrystals that were completely wrapped with AC nanosheets. Owing to the synergetic effects from both nc-Si and AC, a high initial discharge capacity (i.e., 716 mAh/g) and a high reversible specific capacity (i.e., 429 mAh/g) were demonstrated on the AC<nc-Si>AC anode. Furthermore, the AC<nc-Si>AC anode displayed an excellent rate performance and an outstanding cyclic stability with the high coulombic efficiency (~97.5%). The results depict that BRH-derived AC<nc-Si>AC nanocomposites hold huge potential as a high-performance anodic source material for LIB devices.

## Figures and Tables

**Table 1:** Specific surface area and pore characteristics of the BRH-derived (a) nc-Si, (b) AC, (c) AC<nc-Si>AC nanocomposites.

Samples	BET Analysis		BJH Analysis	
	Specific Surface Area (m^2^/g)	Pore Surface Area (m^2^/g)	Pore Volume (cm^3^/g)	Average Pore Diameter (nm)
nc-Si	232.8	153.5	0.2936	5.83
AC	1068.5	755.8	0.4932	2.41
AC<nc-Si>AC	498.5	394.9	0.386	2.45

## Supplementary Materials

The Supplementary Materials are available online at https://www.mdpi.com/2079-4991/9/7/1055/s1, Synthesis Method for Silicon Nanocrystals; Synthesis Method for Activated Carbon; Compositional Properties of nc-Si, AC, and AC<nc-Si>AC.
