# Supplementary material for: Activated Carbon-Decorated Spherical Silicon Nanocrystal Composites Synchronously-Derived from Rice Husks for Anodic Source of Lithium-Ion Battery
Source: Nanomaterials (Basel). 2019 Jul 23;9(7):1055. doi: 10.3390/nano9071055 (PMC6669463; doi:10.3390/nano9071055)
Supplement: Supplementary file 1 [file nanomaterials-09-01055-s001.pdf]

Supplementary Materials

Activated Carbon-Decorated Spherical Silicon Nanocrystal Composites Synchronously-Derived from Rice Husks for Anodic Source of Lithium-Ion Battery

Sankar Sekar [1,2], Abu Talha Aqueel Ahmed [1], Akbar I. Inamdar [1], Youngmin Lee [1,2], Hyunsik Im [1,2], Deuk Young Kim [1,2] and Sejoon Lee [1,2,*]

[1] Department of Semiconductor Science, Dongguk University-Seoul, Seoul 04620, Korea
[2] Quantum-Functional Semiconductor Research Center, Dongguk University-Seoul, Seoul 04620, Korea
* Correspondence: sejoon@dongguk.edu; Tel.: +82-2-2260-3946; Fax: +82-2-2260-3945

■ Synthesis Method for Silicon Nanocrystals

The Si nanocrystals were synthesized from the brown rice husks (BRHs) through the sequential processes of the BRH calcination, HCl treatment, and magnesiothermic reduction. Firstly, through the calcination of BRHs at 500 °C for 2 h under air atmosphere, the BRH ashes were obtained. Then, 3 g of BRH ashes were stirred in 10 % HCl (45 mL) for 2 h to remove the metal ions impurities. Soon after, HCl-leaching, the leached BRHs were filtered, washed, and dried at 150 °C for 12 h in an electric oven. The obtained semitransparent SiO_2 products were subsequently transferred into the alumina crucible, and were annealed at 700 °C for 2 h in a muffle furnace under air atmosphere. Thereafter, the SiO_2 nanopowders were collected by using a centrifugal separator, and were reduced to the nanocrystalline Si (nc-Si) powders *via* the magnesiothermic reaction method. To perform the reduction process, the SiO_2 nanopowders (1 g) were mixed with Mg powders (0.2 g); and then, the mixture powders of SiO_2 and Mg were annealed at 700 °C for 2 h in a tube furnace under Ar atmosphere. Then, the resultant products were stirred in dilute solution of 1 M HCl (HCl: H_2O: EtOH = 0.66 : 4.72 : 8.88 molar ratio) for 8 h to eliminate the Mg_2Si and MgO. As soon as completing the HCl treatment, the products were reacted with 5 % HF for 1 h to remove the residual and/or newly formed SiO_2. Finally, the nc-Si powders were obtained through filtering, washing, and drying processes.

■ Synthesis Method for Activated Carbon

Activated carbon (AC) nanosheets were derived from BRHs *via* the calcination with the KOH activation treatment. Firstly, the BRH ashes were obtained through the carbonization of BRHs at 500 °C for 1 h in air. Thereafter, 4 g of the BRH ashes were mixed with 12 g of KOH to produce the mixture of KOH and BRH ashes. The mixtures were then annealed at 700 °C for 2 h in an encapsulated alumina crucible under air ambiance. Subsequently, the annealed mixtures were stirred in deionized water for 8 h to eliminate the entrapped potassium compounds. Finally, the resultant products were filtered, rinsed, and dried to collect the AC nanosheets.

■ Compositional Properties of nc-Si, AC, and AC<nc-Si>AC

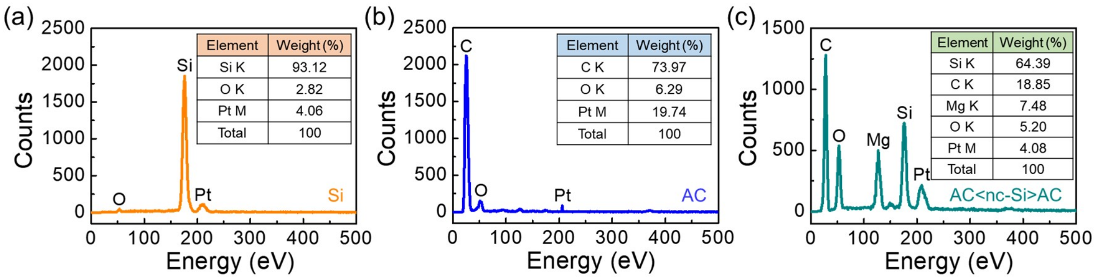

Fig. S1. EDX spectra of (a) nc-Si, (b) AC, and (c) AC<nc-Si>AC nanocomposites. The inset in each figure depicts the tabulated result of main species involved in each product.

From the energy dispersive x-ray (EDX) analysis, the Si nanocrystals, AC nanosheets, and AC<nc-Si>AC nanocomposites are confirmed to possess their main species of Si, C, and Si-C, respectively (Fig. S1). The additional peak from Mg in AC<nc-Si>AC arises from MgO precipitates that were formed during the magnesiothermic reduction process. The presence of Pt peak is thought to emerge from coating of conductive Pt for SEM measurements.

■ Comparison of Electrochemical Performances of Various Si-based Nanocomposites

Table S1. Key LIB Performances of Various Si-based Nanocomposites for LIB Anodes.

Resource	Material	Measurement Condition	Initial Capacity [mAh/g]	Capacity Retention [mAh/g]	Ref.
Rice husk	AC<nc-Si>AC	100 mA/g	716	429 at 200 mA/g after 100 cycles	This Work
Rice husk	Si/C	100 mA/g	1256	537 at 100 mA/g after 200 cycles	[S1]
Rice husk + Commercial	pSi/C/rGO	0.1 A/g	1684	760 at 0.1 A/g after 80 cycles	[S2]
Rice husk + Commercial	Si/rGO	0.05 A/g	1695	830 at 1 A/g after 200 cycles	[S3]
Rice husk + Commercial	Si/C:N/CNT	0.1 A/g	2062	1031 at 0.5 A/g after 100 cycles	[S4]
Bamboo Leaf + Commercial	Si@C/rGO	210 mA/g	2678	1400 at 8.4 A/g after 50 cycles	[S5]
All commercial	Si/G/MW-CNT	100 mA/g	1606	425 at 100 mA/g after 100 cycles	[S6]
All commercial	Si@C	200 mA/g	1713	636 at 200 mA/g after 50 cycles	[S7]
All commercial	Si/rGO/CFP	1 A/g	2844	364 at 1 A/g after 500 cycles	[S8]
All commercial	Si@SiO_x/G	0.1 A/g	2080	1640 at 0.1 A/g after 140 cycles	[S9]

References

S1. Yu, K.; Zhang, H.; Qi, H.; Gao, X.; Liang, J.; Liang, C., Rice husk as the source of silicon/carbon anode material and stable electrochemical performance. *ChemistrySelect* **2018,** *3*, 5439-5444.

S2. Yu, K.; Zhang, H.; Qi, H.; Liang, J.; Liang, C., High performance of porous silicon/carbon/RGO network derived from rice husks as anodes for lithium-ion batteries. *New J. Chem.* **2018,** *42*, 19811-19817.

S3. Jiao, L.-S.; Liu, J.-Y.; Li, H.-Y.; Wu, T.-S.; Li, F.; Wang, H.-Y.; Niu, L., Facile synthesis of reduced graphene oxide-porous silicon composite as superior anode material for lithium-ion battery anodes. *J. Power Sources* **2016,** *315*, 9-15.

S4. Zhang, Y.-C.; You, Y.; Xin, S.; Yin, Y.-X.; Zhang, J.; Wang, P.; Zheng, X.-s.; Cao, F.-F.; Guo, Y.-G., Rice husk-derived hierarchical silicon/nitrogen-doped carbon/carbon nanotube spheres as low-cost and high-capacity anodes for lithium-ion batteries. *Nano Energy* **2016,** *25*, 120-127.

S5. Wang, L.; Gao, B.; Peng, C.; Peng, X.; Fu, J.; Chu, P. K.; Huo, K., Bamboo leaf derived ultrafine Si nanoparticles and Si/C nanocomposites for high-performance Li-ion battery anodes. *Nanoscale* **2015,** *7*, 13840-13847.

S6. Toçoğlu, U.; Hatipoğlu, G.; Alaf, M.; Kayış, F.; Akbulut, H., Electrochemical characterization of silicon/graphene/MWCNT hybrid lithium-ion battery anodes produced via RF magnetron sputtering. *Appl. Surf. Sci.* **2016,** *389*, 507-513.

S7. Zhou, Y.; Guo, H.; Yang, Y.; Wang, Z.; Li, X.; Zhou, R.; Peng, W., Facile synthesis of silicon/carbon nanospheres composite anode materials for lithium-ion batteries. *Mater. Lett.* **2016,** *168*, 138-142.

S8. Tao, H.; Xiong, L.; Zhu, S.; Yang, X.; Zhang, L., Flexible binder-free reduced graphene oxide wrapped Si/carbon fibers paper anode for high-performance lithium ion batteries. *Int. J. Hydrogen Energy* **2016,** *41*, 21268-21277.

S9. Bai, X.; Yu, Y.; Kung, H. H.; Wang, B.; Jiang, J., Si@SiO_x/graphene hydrogel composite anode for lithium-ion battery. *J. Power Sources* **2016,** *306*, 42-48.
